# Characterisation of Biologically Active Hydrolysates and Peptide Fractions of Vacuum Packaging String Bean (*Phaseolus Vulgaris* L.)

**DOI:** 10.3390/foods9070842

**Published:** 2020-06-28

**Authors:** Anna Jakubczyk, Monika Karaś, Piotr Stanikowski, Beata Rutkowska, Magdalena Dziedzic, Ewelina Zielińska, Konrad A. Szychowski, Urszula E. Binduga, Kamila Rybczyńska-Tkaczyk, Barbara Baraniak

**Affiliations:** 1 Department of Biochemistry and Food Chemistry, University of Life Sciences in Lublin, 20-704 Lublin, Poland; barbara.baraniak@up.lublin.pl; 2 Department of Plant Food Technology and Gastronomy, Faculty of Food Science and Biotechnology, University of Life Sciences in Lublin, 20-704 Lublin, Poland; piotr.stanikowski@up.lublin.pl; 3 Scientific Students Group of Food Biochemistry and Nutrition Department of Biochemistry and Food Chemistry, University of Life Sciences in Lublin, 20-704 Lublin, Poland; beata.rutkowska92@mail.com (B.R.); dziedzic.magdalena26@gmail.com (M.D.); 4 Department of Analysis and Assessment of Food Quality, University of Life Sciences in Lublin, 20-704 Lublin, Poland; ewelina.zielinska@up.lublin.pl; 5 Department of Lifestyle Disorders and Regenerative Medicine, University of Information Technology and Management in Rzeszow, 35-225 Rzeszów, Poland; konrad.szychowski@gmail.com (K.A.S.); ubinduga@gmail.com (U.E.B.); 6 Department of Environmental Microbiology, University of Life Sciences in Lublin, 20-069 Lublin, Poland; kamila.rybczynska-tkaczyk@up.lublin.pl

**Keywords:** bioactive peptides, string bean, nutritional potential, vacuum-packing, in vitro digestion

## Abstract

The aim of the study is to characterise biologically active hydolysates and peptide fractions obtained from vacuum-packed string beans (*Phaseolus vulragis* L.) (PB). Unpacked beans were a control sample. The influence on human squamous carcinoma cell line SCC-15 (ATCC CRL-1623) was determined. Packed bean (PB) and unpacked bean (UB) extracts were found to exert no effect on the tongue squamous carcinoma cells. The results of the study indicated that the packing process contributed to the retention of protein, soluble dietary fibre, and free sugar (2.36, 3.5, and 1.79 g/100 d.m., respectively). PB was characterised by higher antioxidant activity (expressed as neutralisation of 2,2′-azino-bis(3-ethylbenzothiazoline-6-sulfonic acid (ABTS ABTS^•+^) and 2,2-diphenyl-1-picrylhydrazyl (DPPH^·^) free radicals) as well as Fe^2+^ chelation and reducing power (IC_50_ = 54.56, 0.46, 3.85 mg mL^−1^; 0.088 A_700/peptide content_, respectively) than the UB samples before hydrolysis. The hydrolysis process enhanced these properties. The IC_50_ value of lipase and α-amylase inhibitory activity of the hydrolysates obtained from UB was reduced. The PB and UB fractions exhibited a certain level of antimicrobial activity against *S. aureus* and *E. coli*. *Candida albicans* were not sensitive to these peptide fractions.

## 1. Introduction

Nowadays, consumers pay more attention to the relationship between diet components and health problems. However, given the ongoing development of civilisation and the pace of life, meals should be not only nutritious but also fast to prepare [1,2]. The food industry searches for techniques that will ensure the highest quality of products and minimally invasive methods of food preservation, as expected by consumers. There are two main trends in the packaging development process related to the protection of the product. One of them is active packaging linked to the production of environmentally friendly renewable packaging materials [3,4]. Another popular packaging method, with a direct influence on the quality of food, also frozen products, is vacuum packaging, where food products are sealed and protected from dehydration or evaporative water loss from their surface. It can also minimise the loss of nutritional compounds [5]. This technique is most often used for the preservation of meat. Currently, it is increasingly being employed for the packaging of other products such as vegetables or fermented plant foods [6]. This method may be the most effective process to retain health-promoting compounds in cruciferous vegetables, compared to, e.g., blanching, boiling, or microwaving. The losses of bioactive compounds during the heat treatment of vacuum-packed products are lower than during traditional cooking, given the leaching of nutrients and other bioactive compounds into the cooking water and the loss of volatile compounds [7,8] during storage of vegetables [9].

Noncommunicable diseases (NCDs), namely, cardiovascular diseases, cancer, and chronic respiratory diseases, are the main causes of death globally. It is estimated that they were responsible for 71% (41 million) of the 57 million deaths recorded globally in 2016 [10]. The risk factors of NCDs include poor diet, unhealthy habits, and physical inactivity. It is known that free sugars contribute to an increase in the energetic value of diets and may cause a positive energy balance, which is the major cause of obesity [11]. Intake of larger amounts of vegetables (containing less free sugars) than fruit is recommended. Pulse crops, e.g., pea, chickpea, lentil, and lupine, and string bean, along with many other types of beans, are the most widely produced vegetables worldwide [12]. They are a rich source of protein, carbohydrates, fibre, and many essential vitamins or minerals. Moreover, their highly nutritious properties have been shown to have beneficial health-promoting effects, i.e., reduction of high cholesterol, prevention of development of type-2 diabetes or cardiovascular diseases, and prevention of various forms of cancer [13,14,15,16].

The results of a study have indicated that vacuum packing as a method for increasing nutraceutical potential cannot be used for all species of vegetables [17]. Therefore, the aim of this study is to characterise biologically active hydolysates and peptide fractions obtained from vacuum-packed string beans (*Phaseolus vulragis* L.) and to determine the impact of vacuum packaging of yellow string beans after in vitro digestion on the nutraceutical potential. String beans are analysed in this study, as they are a rich source of bioactive compounds and are very popular vegetables in many diets. They can be consumed as both processed and fresh food.

## 2. Materials and Methods

### 2.1. Material

Chemical compounds: Hippuryl-L-Histidyl-L-Leucine (HHL), pepstatin A, phenylmethanesulfonyl fluoride (PMSF), α-amylase from hog pancreas (50 U mg^−1^), pepsin from porcine gastric mucosa (250 U mg^−1^), pancreatin from porcine pancreas, bile extract, 2,4,6-trinitrobenzenesulfonic acid (TNBS), 3,5-dinitrosalicylic acid (DNS), p-nitrophenyl acetate (pNPA), starch solution, trypsin, penicillin, streptomycin, phosphate-buffered saline (PBS) without Ca^2+^ and Mg^2+^, hydrocortisone, sodium pyruvate, sodium bicarbonate, fetal bovine serum (FBS), resazurin and dimethyl sulfoxide (DMSO), Mueller-Hinton broth (MHB), Muller-Hinton agar (MHA )were purchased from Sigma-Aldrich (St. Louis, MO, USA). The DMEM/F12 (1:1) medium was purchased from ATCC (Manassas, VA, USA).

### 2.2. Preparation of Yellow String Beans

The yellow string beans were purchased on a local market. Then, 100 g of beans were vacuum-packed into bags made of multilayer olyethylene terephthalate (PET)/cast Polipropylene (CPP) designed for cooking SOUS VIDE and for vacuum packing machines (RM Gastro Polska Sp. z o.o, Ustroń, Poland). The packed (PB) and unpacked string beans (UB) were heated at 100 °C for 10 min. Next, the samples were lyophilised and grounded in a laboratory mill. The powders were stored at −18 °C until further use.

### 2.3. Cell Culture with PB and UB Extract Treatment

The powders of PB and UB were dissolved in PBS (4%, *w/v*). The samples were shaken for 1 h at room temperature and centrifuged at 8000× *g*, 4 °C, for 15 min. The soluble protein in the supernatant was determined according to the Bradford method [18]. The final concentration of PBS in the culture medium was always equal in all experimental groups. The human squamous carcinoma cell line SCC-15 (ATCC CRL-1623) was obtained from the American Type Culture Collection (ATCC, distributors: LGC Standards, Łomianki, Poland). The SCC-15 cells were maintained in DMEM/F12 1:1 medium containing 1.2 g L^−1^ sodium bicarbonate, 2.5 mmol L^−1^ l-glutamine, 15 mmol L^−1^ HEPES, and 0.5 mmol L^−1^ sodium pyruvate supplemented with 400 ng mL^−1^ hydrocortisone, and 10% fetal bovine serum (FBS). The cells were maintained at 37 °C in a humidified atmosphere with 5% CO_2_. They were seeded into 96-well culture plates (Costar, St. Louis, MO, USA) at a density of 6 × 10^3^ (for the 24 h treatment), 5 × 10^3^ (for the 48 h treatment), and 4 × 10^3^ (for the 72 h treatment) per well and initially cultured before the experiment for 24 h. Subsequently, the medium was replaced with a fresh one with rising concentrations (0.0012, 0.0025, 0.0050, and 0.0100 mg mL^−1^) of the extracts (PB and UB).

#### Resazurin Reduction Cell Viability Assay

Resazurin is a water-soluble dye that can be applied in various in vitro cell studies. This method is based on the detection of the metabolic activity of the cell and can be used in metabolism and cytotoxic measurements. The assay was performed according to a method described previously [19]. The cells were exposed to rising concentrations (0.0012, 0.0025, 0.0050, and 0.0100 mg mL^−1^) of PB or UB extracts for 24, 48, or 72 h. After the experiment, the medium in the wells was replaced with the working solution of resazurin (100 µL), and the plates were incubated at 37 °C. The working solution of 60 µM resazurin was prepared in DMEM/F12 medium with 1% FBS from 600 µM stock solution. Fluorescence was measured with an excitation wavelength of 530 nm and emission 590 nm on a FilterMax F5 (Molecular Devices Corporation, San Jose, CA, USA) at 1 h after the addition of the dye.

### 2.4. Enzymatic Hydrolysis

In vitro digestion of string beans (PB and UB) was carried out according to the method described by Jakubczyk et al. [20]. Briefly, lyophilised string beans were grounded in a laboratory grinder, and a 4% (*w/v*) stimulated saliva solution with a final concentration of 7 mmol L^−1^ NaHCO_3_ and 0.35 mmol L^−1^ NaCl, pH 6.75 was prepared. The samples were stirred for 5 min at 37 °C without light. Next, the hydrolysis process was carried out using α-amylase (50 U mg^−1^; the enzyme-to-substrate ratio was 1:10; *w/w*) for 10 min at 37 °C in an incubator without light (Incu-Shaker Mini, Benchmark, Seattle, WA, USA). Next, the pH of the solution was adjusted to 2.5 with 1M HCl and pepsin (250 U mg^−1^) was added (the enzyme-to-substrate ratio was 1:100, *w/w*) and hydrolysis was carried out for 2 h at 37 °C (simulated gastric digestion). For simulated intestinal digestion, the solutions were neutralised to pH 7.0 with 1 M NaOH and supplemented with a 0.7% solution of pancreatin and 2.5% solution of bile extract (1:2.5, *v/v*). After incubation for 1 h at 37 °C without light, the reaction was stopped by heating at 100 °C for 5 min. The hydrolysates were clarified by centrifugation at 8000× *g* for 10 min (MPW, 350R, Warsaw, Poland) and kept at −20 °C.

#### 2.4.1. Peptide Fraction Preparation

Peptide fractions with molecular weight <3.5, 3.5–7.0, and >7.0 kDa were obtained [21] using a membrane tube (MW cut-off 3.5 and 7 kDa) against PBS buffer at the physiological concentration (1:4, *v/v*). The dialysis was carried out without light for 1 h at room temperature. This step was carried out twice. The fractions were collected, lyophilised, and stored at −18 °C until further use.

#### 2.4.2. Potential Bioaccessibility and Bioavailability of Peptides Obtained from String Bean Proteins

Theoretical calculation of the nutritional potential was based on the index described by Karaś et al. [22]. The peptide bioaccessibility index (PAC), which is an indicator of the bioaccessibility of peptides, was expressed as:

PAC = Cph/Cpb,(1)

where Cph—peptide content in the hydrolysate; Cpb—peptide content in the sample before hydrolysis.

The peptide bioavailability index (PAV), which is an indicator of the bioavailability of peptides, was expressed as:

PAV = Cpa/Cph,(2)

where Cpa—peptide content in peptide fractions; Cph—peptide content in the hydrolysate.

### 2.5. Nutrient Analysis

#### 2.5.1. Protein Content

The protein content in nonhydrolysed samples was determined using the Kjeldahl method, and the N conversion factor was 6.25 (CLA/PSO/13/2013). The method is based on AOAC methods [23].

#### 2.5.2. Soluble and Insoluble Dietary Fibre

Fibre was determined before hydrolysis using the enzymatic-weight method (CLA/PSO/2/2011). The method is based on AOAC methods [23].

#### 2.5.3. Reducing Sugar Content

The reducing sugar content was determined before hydrolysis using the DNS method [24].

#### 2.5.4. Peptide Content

The method proposed by Adler-Nissen [25], with L-leucine as a standard, was used. The peptide content was determined in the samples during the hydrolysis process.

### 2.6. Nutraceutical Potential of Hydrolysates and Peptide Fractions

#### 2.6.1. Antioxidant Activities

##### Antiradical Activity (ABTS^•+^)

Antiradical activity against ABTS^•+^ was determined with the method described by Re et al. [26]. The radical solution was prepared with ABTS^•+^ and potassium persulfate, diluted in water at a final concentration of 2.45 mmol L^−1^, and left in the dark for 16 h to allow the development of radicals. The absorbance of the solution was measured around 0.70−0.72 at 734 nm. Then, 0.95 mL of the ABTS^•+^ solution was mixed with 50 µL of each sample. The absorbance was measured at 734 nm after 10 minutes of the reaction. Deionised water was used as a blank.

Scavenging (%) = (1 − (As/Ac)) × 100%,(3)

where As—absorbance of the sample; Ac—absorbance of the control.

All assays were performed in triplicate. The IC_50_ value was defined as an effective concentration of peptide required to scavenge 50% of radical activity.

##### Antiradical Activity (DPPH^•^)

Antiradical activity against DPPH^•^ was determined with the method proposed by Brand-Williams et al. [27] with slight modification. A 50-µL sample was mixed with 0.95 mL of a DPPH solution in 75% methanol. The absorbance was measured at 515 nm, and 75% methanol was used as a control. DPPH radical-scavenging activity was calculated as follows:

Scavenging (%) = (1 − (As/Ac)) × 100%,(4)

where As—absorbance of the sample; Ac—absorbance of the control.

All assays were performed in triplicate. The IC_50_ value was defined as an effective concentration of peptide required to scavenge 50% of radical activity.

##### Fe^2+^ Chelating Activity

The method developed by Decker and Welch [28] was used to investigate the ferrous ion (II) chelating ability of proteins, hydrolysates, and peptide fractions. Briefly, the sample (0.05 mL) was added to 0.02 mL of a 2 mmol L^−1^ FeCl_2_ solution and 0.04 mL of 5 mmol L^−1^ ferrozine. The mixture was shaken vigorously and incubated at room temperature for 10 min. The absorbance was subsequently measured at 562 nm in the spectrophotometer. The percentage of inhibition of ferrozine-Fe^2+^ complex formation was calculated with the formula:

Fe^2+^ chelating activity (%) = (1 − (As/Ac)) × 100%, (5)

where As—absorbance of the sample; Ac—absorbance of the control.

All assays were performed in triplicate. The IC_50_ value was defined as an effective concentration of peptide required for 50% of chelation.

##### Reducing Power (RP)

Reducing power was measured with the method proposed by Hu et al. [29], with modifications described by Karaś et al. [21]. Briefly, 2.5 mL of 0.2 M phosphate buffer (pH 6.6) was mixed with 2.5 mL of 1% potassium ferricyanide (*w/v*) and 1 mL of the sample. The samples were incubated at 50 °C for 20 min, cooled down quickly, and mixed with 2.5 mL of 10% TCA (*w/w*). Next, the mixture was then centrifuged at 15,000× g for 10 min, and 2.5 mL of the supernatant solution was added to 2.5 mL distilled water and 0.5 mL of 0.1% ferric chloride (*w/v*). The absorbance of the solution was measured at 700 nm after 10 min of reaction. An increase in the absorbance of the reaction mixture indicates the reducing power of the sample. Each sample was analysed in triplicates. RP was defined as follows:

RP = A_700_/peptide content of the sample (mg/mL), (6)

where A_700_—absorbance of the sample.

#### 2.6.2. Inhibition of Enzymes Involved in Metabolic Syndrome Pathogenesis

##### Assay of ACE Inhibitory Activity

Preparation of ACE from pig lungs

The angiotensin-converting enzyme was prepared with the method described by Jakubczyk and Baraniak [30]. Lung tissues were homogenised in 0.1 M borate buffer pH = 8.3 containing pepstatin A (0.1 mmol L^−1^) and PMSF (0.1 mmol L^−1^) at 4 °C in a ratio of 1:2 (*w/v*) and centrifuged at 8000× *g* at 4 °C for 20 min. The purification of ACE was initiated by the addition of solid ammonium sulphate at 80% saturation. The sample was dialysed (MW cut-off 12 kDa) for 24 h at 4 °C against 20 volumes of 0.1 M borate buffer, pH = 8.3. The dialysate was centrifuged at 8000× *g* at 4 °C for 20 min, and the supernatant containing the active enzyme was frozen (−20°C) and used for further analysis.

ACE inhibitory activity assay

ACE inhibitory activity (ACEI) was measured using the spectrometric method with *o*-phtaldialdehyde, as described by Chang, Chen, Huang and Chang [31] with slight modifications: the reaction was stopped by adding 0.7 mL of 0.1 M borate buffer with 0.2 M NaOH. The ACE inhibition was determined as follows:

ACE inhibition (%) = (1 − ((A1 − A2)/A3)) × 100%,(7)

where A1 is the absorbance of the sample with ACE and the inhibitor, A2 is the absorbance of the sample with the inhibitor but without ACE, A3 is the absorbance of the sample with ACE and without the inhibitor.

The IC_50_ value defined as the concentration of the peptide that inhibits 50% of the ACE activity was determined by measuring the ACE inhibitory activity and peptide content of each sample.

##### α-Amylase Inhibitory Activity Assay

α-Amylase inhibitor activity (αAI) was measured according to the method described by Świeca et al. [32]. α-Amylase from hog pancreas (50 U mg^−1^) was dissolved in 100 mmol L^−1^ phosphate buffer (containing 6 mmol L^−1^ NaCl, pH 7.0). To measure the α-amylase inhibition activity, a mixture of 0.25 mL of the α-amylase solution and 0.25 mL of the sample was first incubated at 40 °C for 5 min. Then, 0.5 mL of 1% (*w/v*) soluble starch (dissolved in 100 mmol L^−1^ phosphate buffer containing 6 mmol L^−1^ NaCl, pH 7) was added. After 10 min, the reaction was stopped by adding 1 mL of 3,5-dinitrosalicylic acid and heating in a boiling water bath for 10 min. The mixture was then made up to 12 mL with double distilled water. The final results were compared with the activity of the same amount of the enzyme without the inhibitor. The IC_50_ value defined as the concentration of the peptide that inhibits 50% of the α-amylase activity was determined by measuring the α-amylase inhibitory activity and peptide contents of each sample. 

##### Lipase Inhibitory Activity Assay

Lipase inhibitory activity (LI) with p-nitrophenyl acetate (pNPA) as a substrate was measured with the method described by Jakubczyk et al. [33]. Lipase (20 μL, 100 mg mL^−1^) was added to 50 μL of the sample and 1.42 mL of 100 mmol L^−1^ potassium phosphate buffer, pH 7.5. After preincubation at 30 °C for 3 min, the reaction was initiated by mixing the reaction mixture with 10 μL of 100 mmol L^−1^ pNPA solution in dimethyl sulfoxide (DMSO). Changes in absorbance at 405 nm were measured for 10 min. The final results were compared with the activity of the same amount of the enzyme without the inhibitor.

### 2.7. Antimicrobial Activity of Peptide Fractions

The peptide fractions were tested against Gram-positive *Staphylococcus aureus* ATCC 29737 and Gram-negative *Eschericha coli* ATCC 25922 bacteria and *Candida albicans* ATCC 90028 yeast. The strains were obtained from the American Type Culture Collection (ATCC, distributors: LGC Standards, Łomianki, Poland) and stored at 4 °C. All strains were cultured at 37 °C on nutrient broth (NB) medium.

#### 2.7.1. Disc Diffusion Method

The antimicrobial assay was performed using Kirby Bauer’s disc diffusion method according to the Clinical and Laboratory Standards Institute [34] The peptide fractions were dissolved with sterile PBS and filtered through syringe filters (Ø = 0.22 μm). The sterile filter disc (Ø 6mm) was saturated with 10 µL of each peptide fraction at a concentration of 8 mg per disc and placed on MHA with the tested bacteria or yeast. The bacterial and yeast suspension (100 µL) prepared from an overnight culture was adjusted to an inoculation of 10^8^ and 10^6^ CFU mL^−1^ of the bacteria and yeast, respectively. The plates were incubated at 37 °C for 18 h (bacteria) and 48 h (yeast). Discs without samples were used as a negative control. Ampicillin (10 µg/disc) and neomycin (30 µg/disc) were used as positive controls for the bacteria. Antimicrobial activity was evaluated by measuring the inhibition zone (mm) of the tested microorganisms and comparing them with the controls.

#### 2.7.2. Determination of the Minimum Inhibitory Concentration (MIC) and the Minimum Bactericidal Concentration (MBC)

Briefly, 0.5 mL of twofold serial dilutions of each peptide fraction (31.25 to 500 µg mL^−1^) were placed into tubes. Next, 500 μL of the bacterial culture (5 × 10^5^ CFU mL^−1^) were added. The tubes with MHB or bacterial cultures served as negative and positive controls, respectively. Minimum bactericidal concentrations (MBCs) were determined after broth macrodilution by subculturing a sample that showed no microbial growth on the surface of the sterile MHA medium. The plates were incubated at 37 °C for 18 h (MBC). The MBC is defined as the lowest concentration of an antimicrobial agent required to kill 99.9% of the final inoculum after 18 h of incubation.

### 2.8. Statistical Analysis

The data are presented as means ± SEM (the standard error of the mean) of four independent experiments. Each treatment was repeated eight times (*N* = 8) and measured in quadruplicate. The average of the quadruplicate samples was used for statistical analyses. Statistical analysis was performed on the original results. In cell culture experiments, the results were presented as a percentage of the controls. The data were analysed with a one-way analysis of variance (ANOVA), followed by Tukey’s multiple comparison procedure. * *p* < 0.05 vs. the control cultures.

## 3. Results

### 3.1. Effect of Extracts From Pb and UB on Resazurin Reduction in the SCC-15 Cell Line

**Figure 1:**
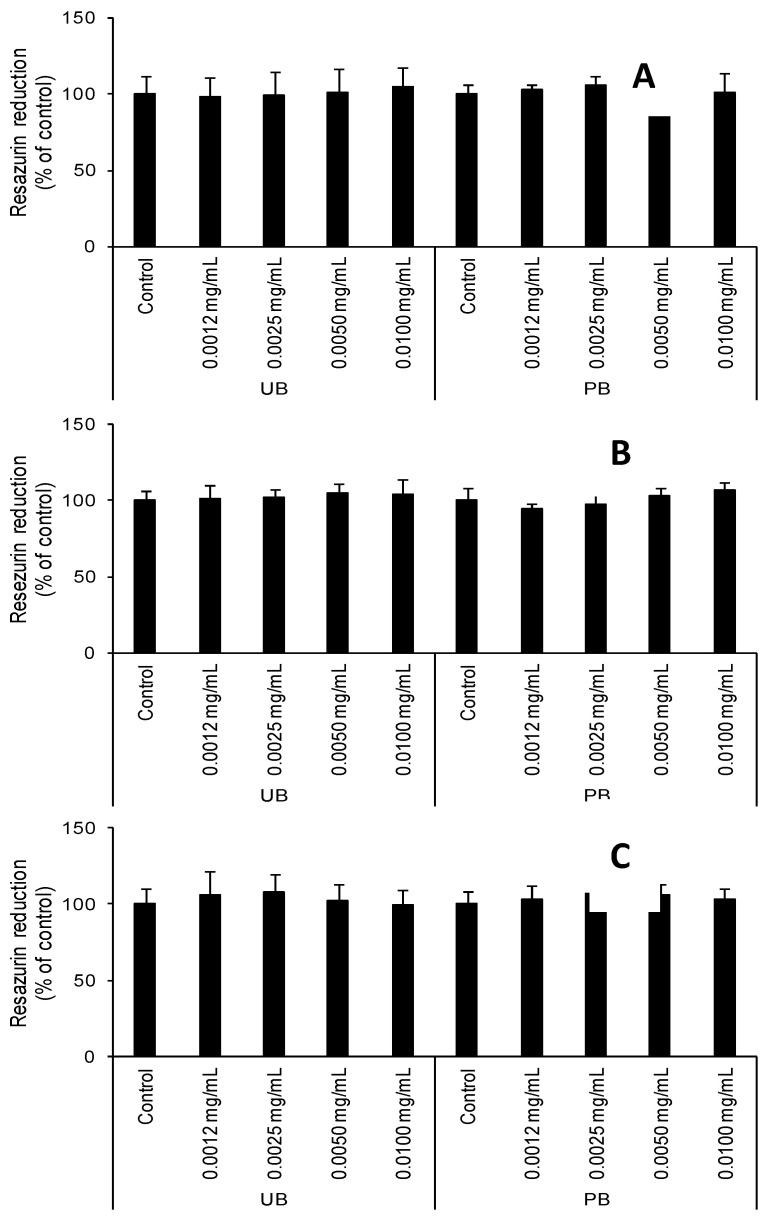
Effect of increasing concentrations (0.0012, 0.0025, 0.0050, and 0.0100 mg) of extracts from the PB (packed string bean) and UB (unpacked string bean) on resazurin reduction in SCC-15 cells after 24 (**A**), 48 (**B**), and 72 h (**C**). The data are expressed as the means ± SEM of four independent experiments, each of which consisted of eight replicates per treatment group. The samples did not show statistically significant differences.

Vacuum packaging of food products may give rise to consumer concerns about the packages used for this process. This was the reason for the determination of the influence of PB and UB extracts on the SCC-15 cell line. After 24, 48, and 72 h of exposure to the studied extracts (PB and UB) at concentrations ranging from 0.0012 to 0.0100 mg mL^−1^, no changes were observed in resazurin reduction compared to the control (Figure 1).

### 3.2. Nutrient Composition of PB and UB

The vacuum packaging process had an influence on the composition of the selected nutrients. As shown in Table 1, the packed string beans were characterised by a higher content of protein (2.36 ± 0.17%), soluble fibre (3.5 ± 0.81%), non-soluble fibre (1.14 ± 0.38%), and free sugars (1.79 ± 0.03 mg mL^−1^) than the unpacked sample. It should be noted that whole pods were used for the analysis; hence, the protein content was low.

### 3.3. Peptide Content during the Hydrolysis Process

**Figure 2:**
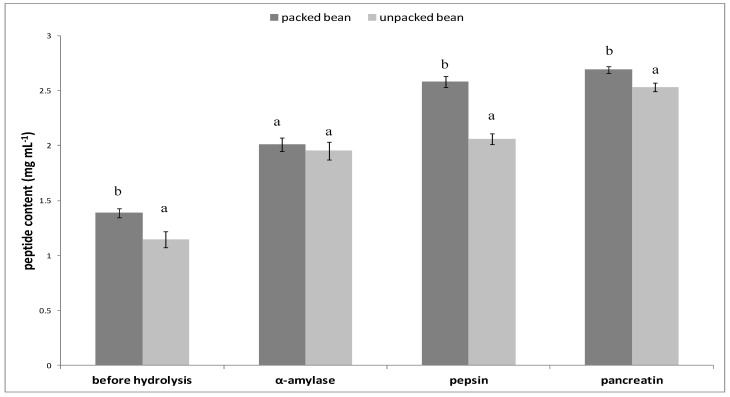
Peptide content in each step of digestion. All values are the mean ± standard deviation for triplicate experiments. The data were analysed with one-way analysis of variance (ANOVA) followed by Tukey’s multiple comparison procedure. Different lowercase letters at the same enzyme indicate significant differences (α = 0.05).

Boiled packed beans (PB) and unpacked beans (UB) were hydrolysed in simulated gastrointestinal conditions. The results show (Figure 2) that the packaging process has an influence on peptides released during hydrolysis. Not only proteases but also α-amylase was used in this process. This enzyme does not cut peptide chains but cleaves starch into smaller carbohydrate groups. In this study, higher peptide content (2.53 mg mL^−1^) was determined for the hydrolysate obtained from PB.

### 3.4. Potential Bioaccessibility and Bioavailability of Peptides Obtained from String Bean Proteins

Besides the peptide content in hydrolysates, there are other important parameters, such as the potential bioaccessibility (PAC) and bioavailability (PAV) of these peptides. As shown in Table 2, the peptide bioaccessibility index for all tested samples was higher than 1. It indicates that the peptides from the packed and unpacked beans are highly bioaccessible in vitro, and the peptides from the unpacked beans (2.21) are characterised by higher bioaccessibility (1.86) than the peptides from the packed beans.

Moreover, the peptides from the string beans had a high potential bioavailability factor. The PAV index was higher than 1 for all peptides obtained from the string beans. It should be noted that the packaging process resulted in an increase in the potential bioavailability of peptides with molecular mass <3.5 and 3.5–7.0 kDa (PAV = 6.04 and 4.21, respectively).

### 3.5. Antioxidant Activity of Hydrolysates and Peptide Fractions Obtained From PB and UB

As shown in Table 3, both the vacuum packaging and the hydrolysis process had an effect on the release of bioactive peptides from the string beans. Of all analysed factors, PB had the highest antioxidant activity determined as IC_50_. Moreover, after separation of the hydrolysates to peptide fractions with different molecular mass, the peptides obtained from PB had the highest antioxidant activities as well. Peptides with a molecular mass lower than 3.5 kDa had the highest antiradical activities against ABTS^•+^ and DPPH^•^ and the highest Fe2+ chelating power (IC50 = 1.07 µg mL^−1^, 0.045 and 0.054 mg mL^−1^, respectively). The packaging and hydrolysis had no influence on reducing power. The same result was noted for the hydrolysates obtained from PB and UB (110 A_700_/_peptide content_).

### 3.6. Inhibitors of Enzymes Involved in the Pathogenesis of Metabolic Syndrome Obtained From PB and UB

The results suggest (Table 4) that both packaging and hydrolysis had an influence on the release of enzyme inhibitory peptides. It should be noted that the hydrolysis process had an effect on peptides with ACE inhibitor activity in particular. Before hydrolysis, this activity was not detected. For all tested activities, the IC_50_ values were lower after the hydrolysis process. In turn, the highest lipase inhibitory activity was exhibited by the fraction <3.0 kDa (IC_50_ = 0.008 mg mL^−1^). Fractions with a molecular mass between 3.5 and 7 kDa were characterised by the highest inhibitory activities against α-amylase and ACE (0.89 and 0.37 mg mL^−1^, respectively). These peptides were obtained from the packed beans. The peptide fraction with molecular mass >7.0 kDa from PB had no α-amylase inhibitory activity.

### 3.7. Antimicrobial Activity of PB and UB Peptide Fractions

The results showed (Table 5) that the PB and UB fractions had certain antimicrobial activity only against *S. aureus* and *E. coli. Candida albicans* were not sensitive to the peptide fractions (Table 5). The results obtained with the disc diffusion method indicated that *S. aureus* was the most sensitive microorganism as it had the largest inhibition zone (13.50–16.30). Lower antibacterial activity was observed against *E. coli* (12.50–15.40 mm). The MIC and MBC values for the tested bacterial strains were in the range of 0.25–1.00 and 0.125–0.50 mg mL^−1^, respectively. Among the tested fractions, UB (peptide fraction 3.5–7.0 kDa) was the most effective bacterial inhibitor and bactericide.

## 4. Discussion

This experiment showed that the vacuum packaging of the string beans had no influence on tongue squamous carcinoma cells. Importantly, the tested extracts did not increase the cell metabolism, as an increase in the cell metabolism in a cancerous cell line would be disturbing. This was the basis for further analysis of bioactive compounds in PB and UB. Moreover, squamous cell carcinomas (SCCs) comprise the majority of malignant neoplasms of the head and neck and represent more than 80% of oral cancers [35]. The World Health Organization (WHO) expects a worldwide rise in the incidence of oral squamous cell carcinomas (OSCCs) [36]. According to statistical data, smokers and alcohol drinkers are a group at high risk of this disease [37]. However, OSCCs also frequently occur in Western societies in nonsmokers and nondrinkers. OSCCs have high mortality and morbidity rates, and in spite of the vast amount of research and the advances accomplished in the field of oncology, the mortality rates remain unchanged [35].Therefore, it seems very important to study the influence of vacuum packing on the quality of string bean, which may affect SCC proliferation and, consequently, accelerate or inhibit the progression of SCC. Protein is one of the most important ingredients of legumes. This compound not only has dietary value but can also be a source of amino acids and peptides released from the protein structure during the hydrolysis process. Afterwards, the peptides are transported into their site of action. The protein content in dry legume seeds is in the range from 22.06 to 32.6%. During the boiling of packed products, nutrient ingredients are not released into the water and removed, but they are retained in the product. In general, fruits stored in modified or controlled atmospheres maintain higher levels of soluble sugars and organic acids, while vegetables show lower values of these compounds [38]. Moreover, vacuum packaging reduces the amount of oxygen present in a sealed package, which prevents the growth of aerobic microorganisms in the stored products. This may also influence nutrient ingredients. There are few studies of the content of nutrient compounds in vacuum-packed products after preparation for consumption, e.g., boiling or baking. The available studies were usually focused on the content of protein and carbohydrates in packed vegetables after storage. Generally, their results indicated that the vacuum packaging process caused the loss of these ingredients, depending on the storage conditions [39]. In contrast, the results in this study have shown that the vacuum packaging process should be used to prepare products that must be cooked before eating and that it protects against loss of components. This is particularly important in the case of vegetables that are rich in protein and fibre, which have a crucial role as bioactive compounds in the organism. The results of our study indicate that the vacuum packing of string bean protects food compounds and reduces the loss of nutrients. Moreover, after hydrolysis, higher peptide content was noted for PB, which may increase the bioactive potential of products.

One of the methods to obtain biopeptides is hydrolysis. In our study, we used the hydrolysis process in simulated gastrointestinal conditions. The peptide content determined in this study corresponds well with the results reported by Karaś et al. [40], where boiled yellow string bean was hydrolysed with pepsin and, after 120 min of the process, the peptide content was 2.5 mg/mL. It should be noted that vacuum packaging ensures the retention of soluble protein in the product, which can be a source of bioactive peptides. Besides the content of peptides, their PAC and PAV indices are important factors. The PAC index for UB and PB was higher than 1, which indicates that the peptides released from the string bean protein fraction were highly bioaccessible in vitro. These results correspond well with our previous study, where peptides obtained from millet protein had a high PAC index as well [22]. It should be noted that the PAV index also indicated that the peptides were generally well bioavailable in vitro. The PAV index in all the tested samples was higher than 1, and the peptides with molecular mass <3.5 kDa obtained from PB were characterised by the highest PAV (6.04). Comparing these results with our previous study, where PAV was lower than 1 for almost all peptides [22], it may be concluded that string bean peptides are potentially highly bioavailable.

Free radicals are produced by aerobic organisms, in particular by vertebrates and humans, in a normal process within the body during respiration [41]. Hence, the generation of these compounds is influenced by oxidation and secondary metabolites, as well as food compounds, drugs, stress, UV radiations, and environmental conditions. Generally, free radicals play different functions in an organism. On the one hand, they can provide defence against infections, but on the other hand, excessive production of free radicals and impaired mechanisms of neutralization thereof can result in cellular damage, which may lead to a number of diseases, especially atherosclerosis, arthritis, diabetes, and cancer. It is important to provide the organism with compounds with antioxidant activities.

The results of the present study indicate that the vacuum packing process enhances bioactive compounds and antioxidant activity, compared to the raw product. The phenolic content in borage after vacuum packing was 1.8 higher, and the antioxidant activity was 2.5 higher than the values noted in the unprocessed plant. Moreover, boiling decreased the total phenolic content by 32%. This process also reduced the color loss of products, which increases the quality of products for consumers [7].

Kosewski et al. [8] determined the antioxidant properties of 22 species of vegetables subjected to conventional cooking and sous-vide methods. The results indicated that the antioxidant activity against DPPH was in the range of 7.47–235 in raw vegetables (μM Trolox/100 g of vegetables), 6.15–657 in vegetables after the conventional cooking process (μM Trolox/100 g of vegetables), and 4.45–648 in vegetables after the sous-vide process (μM Trolox/100 g of vegetables). These results are comparable with those obtained in our study, where the samples after vacuum packing were characterised by higher antioxidant activity than the samples obtained from raw products (Table 3).

The results obtained by Arcan and Yemenicioǧlu [42] showed that the heat treatment of white beans increased the antioxidant activity of their extract. It should be noted that heating caused aggregation of the main protein fraction (albumins) via different mechanisms, e.g., the formation of disulphide bonds, and this process can reduce or mask antioxidant groups. On the other hand, groups with antioxidant activities may be characterised by solubility properties and be released to water during the boiling process. Therefore, the vacuum packaging process can increase the antioxidant activity of compounds contained in string beans.

Metabolic syndrome is a new fatal epidemic mainly affecting the inhabitants of developed countries. They are at increased risk of cardiovascular diseases (CVDs), progression of carotid atherosclerosis, dyslipidemia, central obesity, glucose intolerance, and proinflammatory and prothrombotic states, which reflect underlying insulin resistance and type 2 diabetes. An imbalance in the glycaemic index and a rapid increase in blood glucose may be reduced by the delivery of food compounds inhibiting enzymes involved in the pathogenesis of metabolic syndrome. The main enzymes that play an important role in this process are involved in the metabolism of fats or sugars (e.g., lipase or amylase) and in the process of blood pressure regulation, with the main role played by the angiotensin-converting enzyme (ACE) [43]. This enzyme controls blood pressure, volume, and electrolytes affecting the heart, vasculature, and kidney by converting inactive angiotensin I (decapeptide) to potent vasoconstrictor angiotensin II (octapeptide) [44]. Excessive activity of this enzyme can result in hypertension. Therefore, inhibitors of this enzyme are being searched for use as food ingredients. In this study, we determined lipase, α-amylase, and ACE inhibitory activity.

The results correspond well with those obtained by Akıllıoğlu and Karakaya [45], where common bean digests showed 1.34–2.53 times and green lentil digests showed 10.91–23.65 times higher inhibitory activity against ACE than raw samples. The heat treatment process had a positive influence on ACE inhibitory activity, but it was dependent on the duration of the process. The 15-min heat treatment was the most effective for increasing the ACE inhibitory activity of the stomach digestion. However, in the case of the intestinal digestion of green lentils, all the three heat treatment times brought similar effects. The IC_50_ values reported by Akıllıoğlu and Karakaya [45] were lower than those obtained in this study, which may have been related to the different types of bean or heat treatments and hydrolysis conditions. However, in our study, the IC_50_ values of α-amylase inhibitory activity were higher before the hydrolysis process. The vacuum packaging process had a positive influence on the inhibitory activity. However, there are no data in the literature about the effect of this process on the release of inhibitors of enzymes involved in the pathogenesis of metabolic syndrome. The inhibitory activity was also determined for peptide fractions obtained from the PB and UB hydrolysates. It should be noted that the IC_50_ values were higher for PB than UB only for the ACE inhibitor activity. This indicates that the active compounds should be soluble. Another important property of biopeptides is their antimicrobial activity, which may support the immune system. In this study, we tested peptide fractions against *Staphylococcus aureus*, *Escherichia coli*, and yeast *Candida albicans*. They showed activity only against *S. aureus* and *E. coli.* Our results are consistent with earlier studies, which described the antimicrobial activity of protein hydrolysates and peptide fractions obtained from *P. vulgaris* [46,47]. Oliver-Salvador et al. [46] reported that protein hydrolysates (3–10 kDa) from three *P. vulgaris* varieties showed antibacterial activity against *S. typhi, S. dysenteriae*, and *B. subtilis* but no activity in the presence of *S. aureus* and *E. coli*.

## 5. Conclusions

Vacuum packaging is one of the most common methods for the protection of vegetables against loss of nutrients and bioactive compounds. It may be valuable for the preparation of meals to retain compounds that can be beneficial to the organism, e.g., characterised by antioxidant activity or a capacity to inhibit the metabolic syndrome pathogenesis. The results show that this method can be used to protect bioactive compounds during string bean preparation. Moreover, they also indicate that PB and UB extracts exert no effect on the tongue squamous carcinoma cells. This means the potential safety of the use of vacuum packaging to protect string beans. It should be noted that the packing process contributes to the retention of nutritional compounds such as protein, soluble dietary fibre, and free sugar. Moreover, the packing and hydrolysis process in gastrointestinal conditions enhanced the bioactivities of the samples, which shows that string beans can be used to help treat metabolic syndrome diseases and can also be a source of compounds that inhibit the growth of pathogenic microorganisms.

## Figures and Tables

**Table 1:** Content of selected compounds in packed (PB) and unpacked (UB) string beans.

Select Compounds of String Bean (g/100 d.m.)				
Sample	Protein	Soluble Dietary Fibre	No Soluble Dietary Fibre	Free Sugars
PB	2.36 ± 0.17 ^b^	3.5 ± 0.81 ^b^	1.14 ± 0.38 ^a^	1.79 ± 0.03 ^a^
UB	1.82 ± 0.13 ^a^	2.97 ± 0.98 ^a^	1.20 ± 0.40 ^a^	1.74 ± 0.03 ^a^

d.m.—dry mass; PB - packed sreing bean; UB—unpacked string bean.The data were analysed with one-way analysis of variance (ANOVA), followed by Tukey’s multiple comparison procedure. Different letters in the same column indicate significant differences (α = 0.05).

**Table 2:** Potential bioaccessibility (PAC) and bioavailability (PAV) factors of peptide fractions obtained from PB and UB.

Factor	PB			UB		
PAC	1.86			2.21		
	<3.5 kDa	3.5–7.0 kDa	<7.0 kDa	<3.5 kDa	3.5–7.0 kDa	<7.0 kDa
PAV	6.04	4.21	1.87	1.23	1.27	2.14

PB—packed sreing bean; UB—unpacked string bean.

**Table 3:** Antioxidant activity of samples (IC_50_).

Sample	ABTS (µg mL^−1^)		DPPH (mg mL^−1^)		Fe^2+^ Chelation (mg mL^−1^)		Reducing Power (A_700/peptide content_)	
	PB	UB	PB	UB	PB	UB	PB	UB
Before hydrolysis	54.56 ± 2.16 ^eA^	151.10 ± 18.16 ^cB^	0.460 ± 0.012 ^dA^	1.910 ± 0.07 ^dB^	3.850 ± 0.82 ^dA^	8.880 ± 0.85 ^eB^	0.088 ± 0.002 ^bB^	0.079 ± 0.003 ^cA^
Hydrolysate	22.39 ± 1.17 ^dA^	27.10 ± 1.66 ^bB^	0.110 ± 0.009 ^cA^	0.200 ± 0.007 ^cB^	1.670 ± 0.66 ^cA^	1.760 ± 0.61 ^A^	0.110 ± 0.003 ^cA^	0.110 ± 0.001 ^dA^
Peptide fraction <3.5 kDa	1.07 ± 0.05 ^aA^	7.57 ± 0.76 ^aB^	0.045 ± 0.008 ^bA^	0.063 ± 0.001 ^bB^	0.054 ± 0.003 ^aA^	0.055 ± 0.004 ^aA^	0.083 ± 0.003 ^bB^	0.064 ± 0.002 ^bA^
Peptide fraction 3.5–7.0 kDa	7.6 ± 0.89 ^bA^	7.89 ± 1.09 ^aA^	0.017 ± 0.008 ^aA^	0.053 ±0.002 ^bB^	0.056 ± 0.002 ^aA^	0.072 ± 0.006 ^bB^	0.058 ± 0.013 ^aB^	0.046 ± 0.002 ^aA^
Peptide fraction >7.0 kDa	8.06 ± 0.77 ^cA^	7.79 ± 1.32 ^aA^	0.015 ± 0.004 ^aA^	0.027 ± 0.003 ^aB^	0.150 ± 0.001 ^bA^	0.170 ± 0.004 ^cB^	0.049 ± 0.007 ^aA^	0.076 ± 0.003 ^cB^

All values are the mean ± standard deviation for triplicate experiments. Different capital letters in the same row indicate significant differences for the same indicator (α = 0.05). Different lowercase letters in the same columns indicate significant differences for the same indicator (α = 0.05). The data were analysed with one-way analysis of variance (ANOVA), followed by Tukey’s multiple comparison procedure.

**Table 4:** Inhibition of enzymes involved in metabolic syndrome pathogenesis (IC_50_).

Sample	Lipase (mg mL^−1^)		α-Amylase (mg mL^−1^)		ACE (mg mL^−1^)	
	PB	UB	PB	UB	PB	UB
Before hydrolysis	0.23 ± 0.03 ^dA^	0.23 ± 0.04 ^dA^	2.37 ± 0.19 ^cA^	6.95 ± 0.99 ^bB^	ND	ND
Hydrolysate	0.05 ± 0.008 ^cA^	0.170 ± 0.01 ^cB^	1.54 ± 0.07 ^bA^	1.82 ± 0.05 ^aA^	8.57 ± 1.06 ^cA^	15.73 ± 1.61 ^dB^
Peptide fraction <3.5 kDa	0.008 ± 0.001 ^aA^	0.009 ± 0.0007 ^aA^	13.72 ± 1.11 ^dB^	9.22 ± 0.89 ^bA^	0.84 ± 0.06 ^bA^	1.11 ± 0.08 ^cB^
Peptide fraction 3.5–7.0 kDa	0.023 ± 0.02 ^bB^	0.009 ±0.0009 ^aA^	0.89 ± 0.01 ^aA^	27.31 ± 1.87 ^cB^	0.37 ± 0.009 ^aA^	0.95 ± 0.04 ^bB^
Peptide fraction >7.0 kDa	0.024 ± 0.03 ^bB^	0.013 ± 0.008 ^bA^	ND	10.26 ± 2.88 ^bA^	0.81 ± 0.006 ^bB^	0.59 ± 0.03 ^aA^

PB—packed string bean; UB—unpacked string bean; ND—not determined; All values are the mean ± standard deviation for triplicate experiments. Different capital letters in the same row indicate significant differences for the same indicator (α = 0.05). Different lowercase letters in the same columns indicate significant differences for the same indicator (α = 0.05). The data were analysed with one-way analysis of variance (ANOVA), followed by Tukey’s multiple comparison procedure.

**Table 5:** Antimicrobial activity of peptide fractions.

	Sample	*E. coli* ATCC 25922	*S. aureus* ATCC 29737	*C. albicans* ATCC 90028
	**Inhibition zone (mm)**			
PB	Peptide fraction <3.5 kDa	11.80 ± 1.30 ^a^	14.00 ± 1.00 ^a^	nd
	Peptide fraction 3.5–7.0 kDa	13.00 ± 1.40 ^a^	13.50 ± 1.00 ^a^	
	Peptide fraction >7.0 kDa	12.50 ± 1.00 ^a^	13.5 ± 1.20 ^a^	
UB	Peptide fraction 3.5–7.0 kDa	15.40 ± 1.20 ^a^	16.30 ± 1.50 ^a^	
	AMP	13.50 ± 1.00 ^a^	13.00 ± 1.00 ^a^	na
	NE	23.50 ± 1.50 ^b^	26.20 ± 1.30 ^b^	
	**MIC (mg mL^−1^)**			
PB	Peptide fraction <3.5 kDa	0.50	0.50	nd
	Peptide fraction 3.5–7.0 kDa	1.00	0.50	
	Peptide fraction >7.0 kDa	1.00	0.50	
UB	Peptide fraction 3.5–7.0 kDa	0.50	0.25	
	**MBC (mg mL^−1^)**			
PB	Peptide fraction <3.5 kDa	0.50	0.25	nd
	Peptide fraction 3.5–7.0 kDa	0.50	0.25	
	Peptide fraction >7.0 kDa	0.50	0.25	
UB	Peptide fraction 3.5–7.0 kDa	0.25	0.125	

* values are expressed as the mean ± SD; nd—not detected; na—not applicable; AMP—ampicillin (10 µg/disc); NE—neomycin (30 µg/disc); MIC—minimum inhibitory concentration; MBC—minimum bactericidal concentrations; PB—packed sreing bean; UB—unpacked string bean. Different letters in the same column indicate significant differences (α = 0.05).
